# Modeling Cancer Using Zebrafish Xenografts: Drawbacks for Mimicking the Human Microenvironment

**DOI:** 10.3390/cells9091978

**Published:** 2020-08-27

**Authors:** Pablo Cabezas-Sáinz, Alba Pensado-López, Bruno Sáinz, Laura Sánchez

**Affiliations:** 1 Department of Zoology, Genetics and Physical Anthropology, Universidade de Santiago de Compostela, Campus de Lugo, 27002 Lugo, Spain; pablo.cabezas@usc.es (P.C.-S.); alba.pensado.lopez@rai.usc.es (A.P.-L.); 2 Genomic Medicine Group, Center for Research in Molecular Medicine and Chronic Diseases (CiMUS), Universidade de Santiago de Compostela, 15706 Santiago de Compostela, Spain; 3 Departamento de Bioquímica, Facultad de Medicina, Instituto de Investigaciones Biomédicas “Alberto Sols” CSIC-UAM, Universidad Autónoma de Madrid, Arzobispo Morcillo 4, 28029 Madrid, Spain; bruno.sainz@uam.es; 4 Cancer Stem Cell and Fibroinflammatory Microenvironment Group, Chronic Diseases and Cancer Area 3-Instituto Ramón y Cajal de Investigación Sanitaria (IRYCIS), 28034 Madrid, Spain

**Keywords:** Zebrafish, xenograft, cancer, temperature, microenvironment, chemotherapy

## Abstract

The first steps towards establishing xenografts in zebrafish embryos were performed by Lee et al., 2005 and Haldi et al., 2006, paving the way for studying human cancers using this animal species. Since then, the xenograft technique has been improved in different ways, ranging from optimizing the best temperature for xenografted embryo incubation, testing different sites for injection of human tumor cells, and even developing tools to study how the host interacts with the injected cells. Nonetheless, a standard protocol for performing xenografts has not been adopted across laboratories, and further research on the temperature, microenvironment of the tumor or the cell–host interactions inside of the embryo during xenografting is still needed. As a consequence, current non-uniform conditions could be affecting experimental results in terms of cell proliferation, invasion, or metastasis; or even overestimating the effects of some chemotherapeutic drugs on xenografted cells. In this review, we highlight and raise awareness regarding the different aspects of xenografting that need to be improved in order to mimic, in a more efficient way, the human tumor microenvironment, resulting in more robust and accurate in vivo results.

## 1. From the Problem to the Solution: Cancer and Personalized Medicine 

### 1.1. The Objective of the Modeling: Cancer

Cancer is a term which refers to a wide range of genetically diverse diseases, caused by a de-regulation of the cell cycle, which in turn leads to uncontrolled cell growth and the formation of primary tumors. These abnormal cells acquire specific characteristics that promote their capacity to infiltrate into the blood stream, spread, and reach other tissues forming secondary tumors [1]. This event, commonly referred to as metastasis, is the primary cause of cancer morbidity and mortality [2].

During the last decade, researchers have paid special attention to the metastatic process due to the discovery of circulating tumor cells (CTCs), believed to be the mediators of distant metastases [3]. Briefly, in order to acquire the capacity to intravasate and leave the primary tumor, cells undergo a complex transcriptional reprogramming and change their morphology through a process known as epithelial-mesenchymal transition (EMT), increasing their migratory and invasive capacities [4]. EMT is believed to be a necessary precursor event for intravasation of tumor cells into the blood or lymphatic vessels and their subsequent translocation through the vasculature. Cells which reach secondary target tissues extravasate and suffer the opposite transition (mesenchymal-epithelial transition, MET), giving rise to micrometastases and ultimately to secondary tumors [5,6].

Cancer has become a principal world health problem, representing the second leading cause of death in developed countries [7]. The difficulty of eradicating and effectively treating cancer is multifactorial, but largely due to tumor heterogeneity (both intra and inter-tumoral). Indeed, every type of cancer is different, and even among different patients the same kind of tumor may not be similar (inter-individual heterogeneity), mainly due to the individual genetic landscape and the mutations present across cancer cells [8]. This general heterogeneity is also enhanced by intratumor heterogeneity, which refers to the cellular variability within a tumor, and has been found in the majority of cancer types. Such differences among cells are the product of genome, transcriptome, proteome, and epigenome variations [9]. Altogether, tumor diversity has a key impact on senescence, activation of signaling pathways, migration, invasion, metastasis and importantly, on the response or resistance to treatment [10].

Therefore, efforts have been made over the last decades to dissect the complexity and behavior of different types of tumors, both at the in vitro and in vivo level, to potentiate the development of new and more effective therapeutics. Contrary to in vitro models, the main advantage of using living organisms is that tumor cells and the host directly interact, providing the opportunity to analyze both the tumor and its associated microenvironment, as well as cancer hallmarks, such as metastatic potential, angiogenesis or drug resistance [11,12]. Moreover, in vitro models have been shown to lack clinical predictive power due to the limitations associated with static 2D assays, such as the strong selection pressure associated with cell passaging [13].

Among the different available and accessible in vivo systems, mouse models represent the most commonly used [14]; however, over the last years zebrafish (*Danio rerio*) [15] are increasingly being used for cancer research, specifically in human cancer cell line transplantation (xenograft) and drug discovery assays [16].

### 1.2. A Powerful Tool for Modeling Cancer: Zebrafish

While Zebrafish are very popular aquarium fish, they are also key tools in many laboratories world-wide, with more than 800 biological laboratories using zebrafish as model animals to study different types of diseases [11]. Since the 1960s, zebrafish have traditionally been used as a model for studying developmental biology and vertebrate genetics [17]. More recently, however, the use of zebrafish has been extended to different scientific fields, including human disease modeling [18].

As early as the late 1990s, the advantage of using zebrafish as an animal model to study different diseases was already recognized and since then numerous additional advantages have been identified [19,20]. For example, (1) their transparency during the embryo and larval stage, (2) their size allowing for maintenance of a large number of individual fishes in a relatively small space and (3) the large number of off springs produced daily. In addition, the functional and structural homology that exists between humans and zebrafish is another important advantage, with around 76–82% of the genes involved in human diseases being shared between the two species [21]. With respect to cancer research, the main advantage of the zebrafish is the lack of an adaptive immune system during the first 12–14 days of development [22,23]. Moreover, their transparency during the embryo and larval stage makes visualization of tumor cell growth and metastasis possible, and the similarity between the vasculature of zebrafish and humans allows for neovascularization studies at early stages [24]. The combination of these features also allows for the evaluation of drug response/resistance and anti-cancer agent screening [25]. Lastly, the high number of individual fish that can be used in any given experiment facilitates high statistical power analyses, and at the same time, reduced ethical issues inherent in murine-based studies [13].

The sum of these characteristics make zebrafish an ideal candidate for cancer cell transplantation studies and anti-cancer compound screening assays [26,27].

#### 1.2.1. Modeling Cancer through Xenotransplantation

Xenotransplantation in zebrafish consists of injecting labeled cancer cells into different regions of the zebrafish embryos to track their progression, behavior, and interaction with the microenvironment of the host [28,29].

The first xenotransplantation assay in zebrafish was performed in 2005 by Lee et al., in which researchers used de-differentiated human melanoma cells. These cells were injected in zebrafish blastula-stage embryos and monitored over time. The authors observed the capacity of the human cells to survive, divide and specifically migrate inside the embryos, similar to how melanoma cells distribute in their optimal environment, the skin. These observations supported the idea that zebrafish embryos provided the cells with the necessary signals to specifically integrate into organs, showing bona fide interaction of human cells with the zebrafish microenvironment [30]. Thus, this study demonstrated for the first time the compatibility between human cells and embryos and highlighted the potential value of zebrafish as a biomedical tool for cancer research.

These findings were supported in the following years when Haldi et al. and Nicoli et al. were able to show that after implanting different types of cancer cells in the yolk sac of embryos, cancer cell proliferation, tumor formation, and angiogenesis occurred in zebrafish. These studies were also the first to focus on the specific site of injection, the stage of the embryo, and the incubation temperature, leading to the establishment of the first standardized zebrafish xenograft protocol [29,31].

This protocol established 2 days post-fertilization (dpf) as the most effective stage for xenografting due to the following considerations:

- The zebrafish adaptive immune system is not mature until 4-6 weeks post-fertilization, and during the first 12–14 days of development, only innate immune cells are present. Thus, during the first 2 weeks post birth, induced immunosuppression is unnecessary, and cancer cells can efficiently survive, proliferate, and metastasize in an unaltered host, and even communicate and polarize innate immune cells such as macrophages [22,23].
- Cell tracking is possible due to the transparency of the embryos combined with fluorescence labeling of the cells either via constitutive expression of a fluorescent protein (RFP or GFP), or staining with a lipophilic dye (DiL, DiD, DiO) [26,27].
- Human cancer cells can communicate with the zebrafish embryo cells due to the conserved cell intercommunication shared between these two species. Cell–host interactions, such as the interaction between cancer cells and immune system, are active as can be inferred from neutrophil and macrophage recruitment to the tumor area [32].

**Figure 1:**
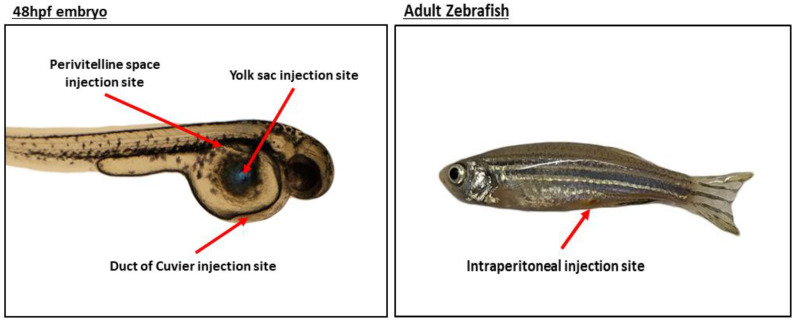
Representative image of the main injection sites.

The standard protocol establishes the yolk sac as the preferred site of injection; although, alternate injection sites have been evaluated and shown to be equally efficient [33,34]. The injection site of choice depends on the cell type and the biological phenotype or event intended to study. The principal sites of injection are listed below (Figure 1):

##### (A) Yolk Sac

Acellular compartment composed by lipids such as cholesterol, phosphatidylcholine, or triglyceride which, when processed and metabolized, provide the fish with energy, ensuring their proper development until they are able to feed themselves, which occurs around 5 dpf [35]. The lipids present represent a source of nutrients for injected human cancer cells, facilitating cell proliferation and tumor growth. In addition, the yolk sac constitutes a delimited space in which labeled cells can be easily visualized over several days. The yolk sac is the site of choice when survival, cell division, proliferation, and migration (if cells are motile) are to be studied [36].

##### (B) Duct of Cuvier (Common Cardinal Vein)

Allows the injection of cells directly into circulation, so different stages of the tumorigenic process can be evaluated in vivo, such as migration, invasion, and MET [37]. In circulation, cells are able to survive, divide, invade, intravasate near the caudal hematopoietic tissue (CHT) (located in the tail of the embryo), and form tumors [32]. Labeled cells can be tracked and proliferation and invasion of tumor cells in the CHT quantified [38]. Cells can also be injected into the perivitelline space to evaluate the efficiency of intravasation [39]. The CHT represents a specific niche favorable for tumor development [40].

##### (C) Perivitelline Space

Located between the periderm and the yolk syncytial layer, the perivitelline space is avascular and not directly communicated with the vasculature region. Aside from proliferation and tumor formation, due to its avascular nature the injection of tumor cells in this site allows for an unambiguous identification of newly formed vessels [29]. In addition, the efficiency of intravasation, further migration, and metastatic behavior can also be evaluated [41,42]. Nevertheless, the performance of xenotransplantantion in the perivitelline space is technically challenging in comparison with other injection sites.

##### (D) Intraperitoneal Cavity

As stated before, injections are usually performed in zebrafish embryos due to the lack of an adaptive immune system. Therefore, assays in adult fish require immune system ablation and, in this situation, injections are performed directly in the intraperitoneal cavity. To facilitate adult zebrafish experiments, an immunocompromised zebrafish line (Rag2 mutant line) was recently created. This line has diminished amounts of T and B cells; therefore, adults can be used without human cancer cell host rejection [43]. Additionally, sublethal γ-irradiation or dexamethasone can be used to induce immunosuppression to allow for cell engraftment in adults, but these approaches are less cost-effective and more time consuming [44,45].

Since the first xenograft assays described by Lee et al. [30], many researchers have improved and refined the technique, developed new strategies and performed xenotransplantations with different cells lines, all the while trying to obtain better, more accurate, and more physiologically- and biologically-relevant results.

#### 1.2.2. Every Powerful Tool Has Its Own Drawbacks

Although many optimal features make zebrafish a suitable animal model for cancer research, as described above, there are some inherent drawbacks, such as incubation temperatures, orthotopic transplantations and cell–host interactions and the microenvironment.

Regarding temperature, while injected human cancer cells should be maintained at 37 °C, the optimal temperature for zebrafish is 28 °C. Thus, a compromise incubation temperature between 28–37 °C should be used, but possible metabolic changes could occur and should be taken into account [46].

Orthotopic xenotransplant consists of the injection of human tumor cells into the site from which they originated (e.g., human brain cells into zebrafish brain). Indeed, while orthotopic injections more reliably mimic the human disease as cells develop in the same anatomical site, and many researchers have successfully performed this technique in zebrafish with retinoblastoma or glioblastoma cells [47,48], this approach cannot be applied to every cell type and in every tissue due to the absence of certain organs in zebrafish, like breast or lung.

Finally, the tumor microenvironment (TME) should be considered in all xenograft assays, irrespective of the host used. The TME refers to the cellular and non-cellular components surroundings and contained within the tumor. Often referred to as the tumor stroma, the TME generally includes cancer cells, non-cancer cells (e.g., fibroblasts, endothelial cells, or immune cells), and extracellular matrix proteins. It represents the site or environment where host and tumor cells interact, playing a key role in tumor growth and progression [49]. In this sense, it is important to consider these interactions in order to mimic the human TME as much as possible and thus, to maximize the full potential of injected cells and allow researchers to study their behavior in the best possible conditions.

## 2. The Cells and the Host: Is There a Perfect Temperature for Both?

As stated above, one of the principal drawbacks of the zebrafish human cell xenograft model remains the temperature limitations associated with the host. Incubation temperature of the embryos has been a subject of discussion since the establishment of this technique in 2005–2006 [30,31]. Zebrafish embryos develop at a temperature of 28 °C in controlled conditions [50], and human cancer cells proliferate and are biologically optimal at 37 °C, the normal physiological temperature in the human body [51]. For this reason, researchers looked for a ‘balance temperature’ between the optimal development of the zebrafish embryos and the human cells injected in initial xenograft studies [29].

In the last years, the standard temperature for performing xenotransplantation assays in the literature has been increased to 34 °C, but at a cost of reducing incubation time to between 3 and 6 days post-injection (dpi) [39,52,53,54,55,56]. This temperature has been chosen in order not minimize harm to the embryos, based on mortality and phenotypic studies [57], but at the same time to achieve a nearly optimal temperature for the injected cells inside the embryo. While a methodological hurdle had been overcome, some experts highlighted the fact that some metabolic pathways could be affected by increasing the incubation temperature of the zebrafish embryos [46].

### 2.1. Finding a Balance between Time and Temperature

Without a doubt, xenotransplantation techniques have evolved over the past decade but little progress has been made with respect to striking a balance between the incubation temperature and the incubation time of injected zebrafish embryos. The outstanding questions of “what happens to the cells or to the embryos if the temperature is raised even more?” and “how many days can embryos be incubated if the temperature is raised?” still remain unresolved.

As mentioned before, the incubation temperature of zebrafish embryos during xenograft experiments should be a balance temperature between the normal development of the embryos under controlled conditions (28 °C) [50] and the optimal temperature of the human cells (37 °C). However, temperature is not considered a lone factor, as it is always linked to the duration of the experiment. In other words, temperature and the time the embryos are exposed to a temperature greater than 28 °C needs to be evaluated and considered together. Various ranges of incubation times with different temperatures have been described in the literature [58,59,60,61], but to date no consensus has been reached. The latter is primarily due to the balance that needs to be struck between not only the temperature of the two components of the experiment (zebrafish embryos and human cells) but the temperature versus the incubation time. What is more important, maintaining the human tumor cells inside the embryos for a long period of time with a lower less human-cell optimal temperature (<37 °C), or incubating the embryos for a short period of time at a higher less zebrafish-optimal temperature (>28 °C)? Some experiments that explore both issues are summarized in Table 1 and explained below.

To address this dilemma in the best possible way, most published studies have followed the standard protocol of 34°C and 6 days of incubation as previously described [39,52,53,54,55,56]. Recently, some studies have specifically modified this ‘standard’ protocol to try and customize it to different experimental settings, depending on what is being assayed or evaluated [59,60,65]. These studies highlight that xenotransplantation techniques cannot be forced to follow a standard protocol, instead, all experimental conditions, including the site of injection, temperature, and incubation time of the injected cells should be tailored to the experimental question and readout, in order to ensure that the most reliable and accurate in vivo results are obtained.

While standard protocols establish the normal incubation temperature at 34 °C, some studies aim to decrease the embryo incubation temperature when performing xenograft assays to 28–33 °C and the time down to 3–4 dpi [62,63,64,65,66,67], increasing the difference in terms of incubation temperature compared to the temperature of the human body, not allowing the cells to perform in their optimal conditions. On the other hand, other studies raise the incubation temperature, increasing the temperature to 35 °C, up from 34 °C [59,61,68,69], or even 36 °C [58], following the suggestion of some authors. It would be interesting to check what happens to the cell proliferation when the temperature is raised [20]. In this case, increasing the incubation temperature of the embryos forces one to decrease the incubation time from 6 days of the standard protocols of 3 dpi due to the mortality caused by the increased temperature. Previous works demonstrate that mortality at 36 °C (12.5%) is not significantly different compared to 34 °C (4.7%) for 3 days of incubation [58]. Assuming these percentages, we consider that for 3 days of incubation it is worth increasing the temperature by 2 °C in order to have a more optimal temperature for the cells while increasing the mortality of the whole experiment to 12.5%.

While all of the aforementioned information should be taken into consideration, we cannot lose sight of the final objective of the zebrafish xenotransplantation technique in the biomedical field: to serve as a useful tool for personalized medicine for cancer patients as ‘avatar’ models [13]. In a clinical setting, the use of an avatar zebrafish model would follow the following scheme: a patient is diagnosed with cancer, a biopsy of the tumor is requested and obtained, the biopsy is cultured or digested in the laboratory to obtain a primary culture or single tumor cell suspension, and cells are injected in a high number of zebrafish embryos, followed by assaying different combinations of chemotherapeutic compounds [18]. Moreover, and in order to increase the biological and clinical relevance of any finding, it would be beneficial to try and mimic, in the best available way, the conditions of the original tumor, considering parameters like temperature [20], site of injection or microenvironment of the tumor [70].

### 2.2. Temperature in the Host: Are the Embryos Suffering Hyperthermia?

As discussed, a balance between the temperature and cell incubation period is critical, but, are embryos physiologically suffering from the temperature increase? One of the crucial components of these in vivo experiments is, precisely, the in vivo host. The zebrafish embryo could be affected due to an increase in the incubation temperature in many ways, including metabolic reactions [46], activation of the innate immune system [71], inflammation [72], or even malformations and increased mortality [57].

The influences that the host exerts upon the injected cells have not been and should be extensively studied. Furthermore, we believe that apart from considering the optimal condition for the injected human cells, it is equally important to check if the integrity of the host, during incubations at temperatures greater than 28 °C, plays or does not play an important role in the proliferation and dissemination of the xenografted cells.

#### 2.2.1. Is the Morphology and Mortality of the Embryo Influenced by Temperature?

Malformations and disruptions in the normal development of the zebrafish embryo due to different incubation temperatures have been studied since the 1960s, but mostly in the first stages of development, from fertilization to the blastula stage [73] due to the fact that zebrafish has been a model organism for developmental genetics [17]. In recent years, teratogenic effects of temperature on embryos have been described in different fish species [74], and, at least to our knowledge, only one study by Pype et al. has described teratogenic effects in zebrafish embryos, specifically mortality and malformation as a consequence of high temperatures [57]. Other studies, however, have focused on the effect of temperature on the immune system, circadian clock or heat-induced masculinization or morphometric traits [75,76,77]. Together, these studies performed with high temperatures provide important information regarding different aspects of the biology and physiology of zebrafish, including mortality or malformations, at high temperatures, but none of them have evaluated heat-induced effects in xenograft assays and how temperature could be influencing the whole state of the host and the reaction to the injected human cells.

Malformations due to heat-induced teratogenic effects could be a consequence of transcriptomic changes in the embryo, modifying morphological aspects, like hatching rates, spine curvature, or edemas [57]. While some authors have reported several malformations, like spinal deviation, edemas, and coagulation of the embryos at a temperature of 32.5 °C, these experiments were performed from fertilization to 96 h post-fertilization (hpf), covering the most critical time point of embryonic development (0 hpf to 48 hpf) [57,75]. Hence, this information is not directly transferable to embryos incubated at 34 °C or more from 48 hpf after hatching, to 3–6 dpi. Reviewing the xenograft literature using zebrafish embryos, even when fish were incubated at 35 °C [59,61,68,69] the malformations highlighted by Pype et al. [57], were not visible in imaged embryos. Apart from the different initiation and end time point conditions between the studies, and excluding the most critical developmental stage of the embryos, the lack of reported or documented malformation incidence could be due to the selection of the embryos without malformations when the first images of injected cells in the xenograft assays were performed. Thus, by purposefully selecting those embryos with the best integrity and highest probability of survival without malformations up until the last day of incubation, previously published studies may have introduced a bias in order to take a reference to study the malformations and mortality in these cases.

Long et al. performed temperature response assays (16 °C and 34 °C compared to a 28 °C control), starting at 96 hpf in zebrafish, with incubation times of 2 h and 48 h [46]. This study is closer to the temperature range of the xenograft assays (48 hpf to 120 hpf or 192 hpf) and is therefore useful as a point of reference. The main conclusions of the study regarding embryo morphology at 34 °C for 48 h was that there were no significant differences between 34 °C and 28 °C, with the parameters analyzed. Nevertheless, there was a change in the dry mass of the embryo, with a reduction at 34 °C, consistent with accelerated development when the embryos are incubated at higher temperatures [46].

#### 2.2.2. How Are the Embryos Reacting to the Temperature at the Transcriptomic Level?

In addition to the study of malformations and mortality rates produced in the zebrafish embryos, another question remains unanswered: how is the embryo reacting to this temperature at the transcriptomic level? Are they compensating for the temperature excess through the upregulation or downregulation of different pathways that could interfere with the injected human cells?

There are numerous studies in the literature that focus on the transcriptomic changes that zebrafish embryos overcome due to increases [78] or decreases in incubation temperature [79] at different points of development. Most of these studies have focused on the crosstalk between cold acclimation in zebrafish larvae and hypoxia [80] or, between the increase in incubation temperature and hypoxia [81]. Even if those studies were not performed in the time-window used in the xenograft assays, the transcriptomic information they provide is useful to elucidate some of the pathways involved or affected by temperature stress, especially when the temperature is increased.

While, it has been shown that zebrafish embryos and larvae are resilient to death and malformations with incubation temperatures up to 31 °C in their first week of life, the combination of additional different stressors (like hypoxia, apart from temperature) across different developmental stages of the zebrafish could result in decreased resilience [78]. This is confirmed when the communication between the pathways related to heat stress and hypoxia are studied at the gene expression level [81]. These studies provide valuable information regarding the influence between the heat stress and hypoxia to improve the survival rates of xenografted zebrafish embryos exposed to high temperatures, and to study the combination of factors (injection site, temperature, density of fish per area, dissolved oxygen, etc.) that could be affecting the embryo in order to reach a more optimal condition for the xenografted human cells.

In other studies, authors have tried to assay the overall molecular mechanisms underlying the temperature acclimation of zebrafish embryos by microarray assays, instead of focusing on particular pathways [46]. In this study by Long et al., microarray data showed an upregulation and downregulation in different genes and pathways during the incubation of zebrafish for 2 h and 48 h at different temperature conditions (16 °C, 28 °C, and 34 °C). Focusing on the 34 °C condition, the authors showed an increase in gene expression when the incubation time was increased to 48 h. Among the upregulated genes, genes involved in processes related to development (lipid catabolism or oxidation-reduction processes) were identified, but more importantly, a large number of immune system-related genes were modulated [46].

Thus, if the incubation temperature is increased during the experiment, changes in host gene expression as a result of temperature acclimation are certainly occurring and maybe, indirectly, the cells injected inside the zebrafish are being modified as a consequence of these parameters and transcriptional responses. Of all the multiple transcriptional changes occurring in the zebrafish embryos when the temperature is increased, changes observed in immune-related genes could significantly influence the injected human cells. Zebrafish embryos start to develop their adaptive immune system between 12–14 dpf, and the complete maturation of the adaptive immune response is achieved between 4 and 6 weeks post-fertilization [22]. This is one of the main advantages of this model as embryos do not require immunosuppression for performing xenograft assays [20], unlike mouse-based systems that depend on genetically modified immunocompromised mice. While lacking an adaptive immune system, zebrafish embryos possess an innate immune system provided by the mother, consisting in macrophages and neutrophils that are distributed all over the embryo at 48 hpf, when the xenograft assays take place [55,82]. Thus, under different stimuli and stress responses from the environment (e.g., injected cancer cells, increased temperature, or bacteria [71]), the host innate immune system can react and modify the inflammation landscape of the embryo [72], among other pathways [46], and indirectly affect the xenograft.

We already have some idea of what is happening with the innate immune system in zebrafish embryo xenograft assays. Some studies have focused on the interaction of macrophages with injected human cells, and have demonstrated an interaction between macrophages and the angiogenic response towards the tumor [83], while other authors have studied neutrophils and their role in tumor progression. Interestingly, it has been shown that zebrafish neutrophils play a crucial pro-metastatic role, with neutrophils accompanying breast cancer cells in circulation, facilitating their migration and invasion to metastatic niches [84]. While we have some clues regarding the role of zebrafish innate immune cells during the xenograft process, there is still a lack of studies related to how temperature modulates these immune cells, as mentioned above. Likewise, temperature-mediated changes are not solely limited to the immune system (innate immune response and inflammation), and could also be involved in different pathways related with the response of the host (e.g., metabolism), in this case the zebrafish embryo, to the human cells injected, altering the results obtained from the xenograft at multiple levels. Thus, more studies are needed to fully understand the impact that temperature (and other external stimuli) may have on the physiology, biology and omics of the zebrafish, and how those changes can impact experimental results.

### 2.3. Chemotherapeutic Compounds Trials: Does Temperature Matter?

We have discussed the effects of temperature on the injected human cells and the zebrafish embryo host, but one concern still remains: do the aforementioned heat-induced changes affect the activity or metabolic processing of chemical compounds, such as anti-cancer drugs?

In the personalized medicine field, a main objective is to use zebrafish embryos as an avatar compound-screening model in order to provide a useful, low-cost and fast tool to perform personalized medicine studies to help oncologists make rationalized decisions and determine the optimal treatment for each patient [13,18]. This is one of the main advantages of this animal model compared to murine models, the capacity to perform ‘high-throughput’ screening of compounds in as little as one week with significantly reduced amounts of compounds [85,86]. Thus, the speed and overall reduced costs of zebrafish avatar models would allow for its implementation as a tool for personalized medicine in the cancer clinical setting [87]. However, and related to the main drawbacks already presented, temperature could be influencing drug screening results. Temperature conditions are normally established around the 31–34 °C to ensure both ‘normal’ cells growth and that the zebrafish embryos survive [88]. However, if cell proliferation is reduced at lower temperatures (34 °C or lower) and the chemotherapeutic agent tested targets highly proliferative cells via DNA base intercalation, like 5-Fluorouracil [89], then compounds like 5-Fluorouracil may show reduced efficacy. Therefore, cells must be incubated at a temperature where replication is not affected, otherwise the effect of such compounds will not be detected in vivo [58]. In order to avoid an over or under estimation of a compound’s effect, and considering that temperature could be influencing the proliferation of the injected human cells inside the zebrafish embryo, different authors have already suggested that a temperature closer to 37 °C (physiological temperature of the human body) is desirable in zebrafish-based drug screening assays [20].

## 3. Microenvironment

As mentioned before, the tumor microenvironment is composed of cellular and non-cellular components, producing a constant interaction with the host, controlling cell proliferation at the primary site, and facilitating the dissemination of cancer cells and their subsequent colonization of other tissues. The principal cellular components of the tumor microenvironment are cancer-associated fibroblasts (CAFs), endothelial cells and pericytes and tumor-associated macrophages (TAMs) [49,90].

### 3.1. CAFS, Endothelial Cells, and Perycites

CAFS derive from normal stroma fibroblasts, which are stimulated by tumor cytokines or factors, including transforming growth factor-β (TGF-β). They are mainly involved in promoting proliferation and migration through the activation of surface markers like fibroblast-activating protein (FAP) [91], and secretion of signaling molecules and cytokines such as epithelial growth factor (EGF) or insulin-like growth factor (IGF-1). CAFs also contribute to tumor progression by remodeling the extracellular matrix through the production of collagen and fibronectin [92]; enhancing cell migration and invasion by degrading matrix enzymes [93]; and increasing cancer cell motility and invasiveness through the generation of pro-invasive and angiogenic molecules like vascular endothelial growth factor (VEGF) and interleukin 6 (IL-6) [94,95].

Pericytes have the ability to stimulate the proliferation and migration of endothelial cells [96]. In turn, endothelial cells have the ability to recruit bone marrow-derived endothelial progenitor cells and promote the formation of new vasculature [97] as well as secrete factors which control leukocyte recruitment and metastasis [98].

### 3.2. TAMs

Not only are TAMs the most abundant cell type in the tumor microenvironment, but TAMs have become an increasingly interesting target for cancer therapy [16]. Macrophages are myeloid immune cells located in every body tissue involved in physiological processes such as innate immunity and inflammation. They can be simply classified according to their activation/polarization towards the classical pro-inflammatory or the alternate wound-healing state, [99] which are both involved in the initiation or resolution of inflammatory processes, respectively. M1 macrophages have been classically named as inflammatory macrophages, while M2 are known as wound-healing macrophages [100].

The M1 phenotype can be adopted in response to the secretion of cytokines such as interferon-*γ* (IFN-*γ)* or tumor necrosis factor-*α* (TNF-*α*) or by pathogen-associated signals. This activation leads to the production of reactive nitric and oxygen intermediates promoting a cytotoxic and anti-proliferative activity, and to an inflammatory response through interleukins like interleukin-1 (IL-1) or interleukin 12 (IL-12) and TNF-*α.* On the other hand, macrophages can be polarized to an alternatively activated (M2) state by stimulation with interleukin-4 (IL-4), interleukin- 10 (IL-10), interleukin-13 (IL-13), or glucocorticoids. This macrophage phenotype can produce angiogenic mediators, such as TGF-β, VEGF or EGF, which also mediate inflammation resolution and an immune suppressive environment [101,102,103].

Cancer cells secrete cytokines and chemokines promoting the recruitment of macrophages to the tumor and converting these immune cells into the main inflammatory component in the tumor microenvironment, thus receiving the name of TAMs [102]. Tumor cells are able to modulate the activity of recruited macrophages and shift these cells to display an M2-like phenotype [104]. Nevertheless, M1-like macrophages are more common in tissues where tumors have recently started to develop, and the phenotype switch to the M2-like state occurs when the tumor begins to progress, vascularize, and invade [105]. Thus, TAMs are able to perform a variety of activities within the tumor microenvironment, promoting several key tumor processes, such as metastasis, immune inhibition, or angiogenesis.

Regarding metastasis, the ability of TAMs to facilitate progression and invasion implies reciprocity between these cells and cancer cells. Within the tumor microenvironment, recruited TAMs produce EGF to which tumor cells respond through the epidermal growth factor receptor (EGFR) present on their cell surface, enhancing cell invasion and migration [106]. In turn, cancer cells express colony stimulating factor 1 (CSF-1), also known as macrophage stimulation factor-1 (M-CSF), a powerful chemotactic molecule for TAMs, which express the colony stimulating factor 1 receptor (CSF1R) [103]. In addition, TAMs are able to remodel collagen fibers and carry tumor cells to the proximity of blood vessels, facilitating the intravasation into the vasculature [107].

With respect to immune inhibition and, as mentioned above, TAMs present an M2-like phenotype and thus, they are able to develop an immune suppression response through the secretion of immune suppressive molecules like TGF-β, arginase-1 or nitric oxide (NO), leading to a T-cell response blockade against tumor antigens [108,109]. TGF-β also blocks the stimulation, proliferation, and effector functions of CD4+ and CD8+ T-cells [110]; arginase-1 acts as an inhibitor of arginine, which is necessary for conventional T-cells to be activated [111]; and NO has a synergetic effect with arginase-1 [16].

Finally, concerning angiogenesis, a vascularized state with newly formed blood vessels (angiogenic switch) is required for tumor growth and expansion [112]. The ability to promote angiogenesis is related to a subpopulation of TAMs, which express a type of tyrosine kinase receptor called TIE2. These cells are known as Tie2-expressing macrophages (TEMs) [113]. TIE2 is a receptor for angiopoietins, which are growth factors required for the formation of blood vessels [114].

### 3.3. Mimicking the Tumor Microenvironment

It has been demonstrated that zebrafish xenograft models allow for the study of many of the cancer hallmarks, including tumor progression, angiogenesis, dissemination, metastasis, or drug responses [115]. However, considering the influence of the tumor microenvironment on tumor progression, the interactions between cancer cells and the cellular components of the microenvironment should be taken into account. In doing so, studies may better and more accurately mimic the tumor environment and provide improved opportunities to develop personalized medicine approaches.

#### 3.3.1. Interaction between Tumor and Immune Cells—Zebrafish Transgenic Lines

An important first step to creating more complex zebrafish avatar models would be to use models that allow for the evaluation of the interaction between human malignant cells and the immune cells of the host, mainly neutrophils and macrophages, as they are often supportive of tumor progression and metastasis, as described above. Equally important is angiogenesis, one of the first processes studied using zebrafish xenotransplantation models. Thus, several studies using specific ZF lines have been developed towards these ends.

First, there exists reporter zebrafish lines, such as *Tg(mpx:GFP)^i114^* [116] for neutrophils or *Tg(mpeg1:eGFP)^gl22^* [117] and *Tg(mpeg1:mCherry)^UMSF001^* [118] for macrophages. In order to further characterize the potential that tumor cells have to develop new blood vessels and in turn, their ability to invade and form micrometastases, several transgenic lines in which the vasculature is labeled are also available; *Tg(fli1:eGFP)^y1^* [119]; *Tg(flk1:eGFP)^s843^* [120]; *Tg(flk1:mCherry)* [121]. By using these reporter lines and stereo or confocal microscopy, immune cells, live vessel formation and individual cell growth can be easily detected and monitored in real time [24].

When tumor cells are injected directly into blood circulation through the distal branch of the duct of Cuvier or in the perivitelline space, proliferation and invasion of tumor cells is observed in the CHT [38]. Immune cells are then recruited to the tumor area in the CHT and thus, the number of neutrophils and macrophages, which infiltrate and surround the tumor site indicates the interactions between cancer and immune cells [32].

A pioneering study with macrophage, neutrophil, and vasculature zebrafish reporter transgenic lines and different tumor cells lines was performed in 2012 by Snaar-Jagalska and colleagues, in which the authors showed the involvement of such immune cells in tumor vascularization and invasion. These immune cells were recruited to and localized with tumor cells both in the site of primary tumor growth and at micrometastasis sites. In addition, the authors observed that the non-disseminated tumor cells associated and remodeled the endothelial cells of the duct of Cuvier into structures similar to neovessels, which subsequently formed functional vasculature. To further support this observation, they used antisense oligonucleotides (morpholinos) to transiently knockdown a transcription factor which controls the development and differentiation of myeloid cells, and they were able to suppress tumor vascularization, invasion and micrometastases [39]. These same results were also obtained in a more recent study by Roh-Johnson et al., where the authors showed a dynamic interaction between immune and cancer cells [122]. Specifically, the authors showed that macrophages transfer their cytoplasmic contents to tumor cells in zebrafish and mouse models, and this content exchange correlated with melanoma cell dissemination. While the authors did not identify the exact factors provided by the macrophages, they hypothesize that “motility machinery”-related mRNAs provided by the macrophage are used by the cancer cells to facilitate their directionality and persistence in vivo [122]. In another study, Hill et al. used a vasculature reporter transgenic line in which they injected melanoma cells and recorded, using time-lapse microscopy, how these cells were able to migrate from the site of injection (yolk sac), interact with the endothelium of the blood vessels and form secondary tumors, in a single-cell manner [40].

Britto et al. have also focused on studying the role of innate immune cells during tumor angiogenesis in zebrafish. They established xenografts in a vasculature reporter line injecting several types of cells into the perivitelline space and they quantified vascularization by live-imaging, confirming the ability of tumor cells to induce angiogenesis. They quantified the amount of VEGFA secreted by the cells lines and found a positive correlation between the most vascularized tumors and higher secretion of VEGFA [123]. Importantly, they also evaluated the role of macrophages in vascularization, and using a macrophage reporter line, found an interaction between macrophages and blood vessels, and a positive correlation between the number of immune cells recruited to the tumor site and the degree of angiogenesis [123].

These and other studies strongly support that use of vasculature, macrophage, or neutrophil reporter zebrafish lines to better understand how tumor cells interact with the immune cells and vasculature of the host, and thus, the implication of such interactions in tumor behavior, development, and metastasis. These models allow for non-invasive live imaging of tumor cell progression, migration, tumor-induced angiogenesis, and tumor cell–host cell interactions at a single cell level in a short period of time [124], approaches difficult to carry out in standard mouse models. In addition, the information that researchers can obtain from zebrafish reporter line assays could enable them to develop more accurate drug screening approaches and increase their understanding of a patients’ tumor or response to treatment [20].

#### 3.3.2. Mimicking the Tissue Niche—Orthotopic Xenografts

In recent years, however, orthotopic transplantations in zebrafish have become increasingly popular, as they represent a step closer to more faithfully recapitulating the complete human tumor microenvironment. As previously mentioned, an orthotopic xenograft consists in the implantation of tumor cells into the site/organ equivalent to the origin of the tumor, as a means of more reliably mimicking the patient’s original tumor microenvironment [125]. In doing so, the tumor that forms will contain not only the cellular components of the patient’s original tumor, but also the non-cellular environment provided by the specific site and/or organ. The latter refers primarily to the extracellular matrix (ECM) and its related molecules, and physical and chemical parameters, such as pH or interstitial pressure [49]. It has been shown that the ECM is a dynamic element of the tumor microenvironment and perturbations of ECM-related molecules or metabolites are able to vary cell proliferation, migration, angiogenesis or metastasis [126,127]

Within the last years, several orthotopic xenograft zebrafish models have been developed and in combination with the above-mentioned transgenic lines, many of them have highlighted their recapitulative potential and clinical relevance [128]. The vast majority of orthotopic xenograft studies have been performed with brain tumor cells. For example, one of the first brain orthotopic studies was carried out by Lal et al., using glioblastoma, the most common and aggressive primary malignancy of the central nervous system, as the tumor model [129]. The authors performed xenografts both in the yolk sac and in the brain and found important differences in the behavior of the tumor cells. While they could observe how transplanted cells invaded the brain and also dispersed along the surface of blood vessels, those injected in the yolk sac were neither able to significantly increase in number or invade surrounding tissues [130]. These results are consistent with the fact that the yolk sac lacks the myelinated tracts of axonal surface, which represents, together with the blood vessels, paths for dispersion of glioblastoma cells in the brain [131]. Successive studies similarly showed how cells can proliferate, invade the brain parenchyma, and interact with blood vessels only when injected orthotopically. Moreover, additional technical advances have also led to a better understanding of tumor cell behavior in the zebrafish brain, for instance, the introduction of time-lapse confocal microscopy and novel methods for quantifying tumor progression and cell interactions in 4D [132]. These results point to the zebrafish as a more than accurate animal model to investigate tumor progression, migration, angiogenesis, and the influence and role of the tumor microenvironment. Furthermore, zebrafish orthotopic brain xenografts offer the notable advantage of performing high-throughput drug screenings and, at the same time, studying the ability of potential drugs to penetrate the blood–brain barrier [133,134,135]. Alternative strategies have also been developed to allow for long-term study of tumor cell behavior and drug response by the transplantation of zebrafish-derived tumors into immune-competent hosts [136]. For example, Casey et al. described an orthotopic implantation method where pediatric zebrafish brain tumors were injected into 2-day-old zebrafish host embryos to study tumor cell behavior and drug response over many months [136]. This alternate syngeneic approach could be applied and extended to other zebrafish tumor types.

Orthotopic xenograft zebrafish studies have also been performed to study retinoblastoma, the most common pediatric intraocular cancer [137]. In the most recent studies, the authors injected retinoblastoma cells intravitreally and observed how the cancer cells were able to disseminate outside the eye, and how spread was reduced when fish were treated with different inhibitors to block transcriptional pathways, potentially related to the development of the disease. These results may have translational value, opening the door to the discovery of new therapies for retinoblastoma [138,139]. Additionally, a novel orthotopic xenograft model for conjunctival melanoma has been recently established by retro-orbital injection, to mimic primary tumor spread and to test the possible repurposing of the anti-tumor ruthenium-based photosensitizer TLD1433 for retinoblastoma [124].

Despite the benefits that orthotopic transplantation offers, it is worth noting, as stated above, that in the zebrafish not all human organs are present (e.g., breast or lung), so this technique, although accurate in many cases, is not applicable for all tumor types. However, it has been suggested that this limitation could be overcome by means of using analogous structures, such as the gills as a substitute for lungs [20].

Although the different strategies mentioned above can be used to better understand the interaction between tumor cells and the host and its microenvironment, patient tumor heterogeneity was not the end goal of these studies and some of the approaches described above may not be feasible to study tumor heterogeneity [40]. Thus, in the last decade the patient-derived tumor xenograft (PDX) has emerged as an important technique in cancer research.

#### 3.3.3. Addressing Intratumoral Heterogeneity—Zebrafish PDX (zPDX)

Due to the genetics and occurrence of mutations in cancer cells, the disease is generally very heterogeneous across individuals, and thus a one drug fits all approach for treating cancer has not been successful. For this reason, the last two decades has seen increasing efforts to develop personalized medicine strategies and approaches to better treat patients rather than tumors [140]. In order to preserve the integrity and heterogeneity of the in vivo tumor microenvironment [141], and considering the invaluable characteristics of the zebrafish, researchers have recently started to utilize zPDXs. Briefly, this technique consists in the isolation of fragments or cells from the primary tumor human tissue, which are subsequently injected into the zebrafish. This model can be divided into two categories depending on the site of injection; heterotopic or orthotopic zPDX.

The use of patient-derived cells represents an advancement compared with classical xenograft assays that have depended on laboratory established stable cancer cell lines, which, in most cases, differ dramatically from primary patient-derived tumor cells or tumor pieces [40]. Primary cultures preserve the original phenotypes and features of the tumors of origin, which is essential for the reproduction of the microenvironment [142] and additionally, these cultures also preserve stem-like phenotypes which reaffirm them as a valuable preclinical tool to anticipate patients´ response to treatment, as it is known that cancer stem cells (CSCs) play an important role in drug resistance mechanisms [143]. Consequently, zPDXs theoretically could recapitulate the tumor diversity and biology, maintain the gene-expression and mutational profile of the original tumor, and accurately predict a patient’s potential medical outcome and tumor chemo sensitivity profile [144]. zPDX were pioneered by Marques et al., in 2009, where tissue fragments or cell suspensions from colon, pancreas, and stomach primary tumors were transplanted into the yolk sac of zebrafish embryos. The authors observed cell invasion and metastasis formation in all samples, establishing a robust in vivo model for studying and modeling tumor cell invasiveness and the metastatic behavior of human primary tumors [145]. Since then, several researchers have shown the applicability of the zPDX models for translational research. In some of the notable zPDX models summarized in Table 2, researchers have adapted their approaches in order to overcome technical issues and improve the reliability of their results. For example, Bentley et al. made an unprecedented technological advancement as they were the first to establish a prolonged engraftment of T-cell acute lymphoblastic leukemia cells from two patients, determine the mutational status and correlate the mutations with the response to treatment [69]. Mercatali et al. showed that xenografted breast cancer patient-derived bone metastatic cells have bone marrow tropism after the injection in the duct of Cuvier. As such, cells were able to survive, extravasate, and engraft in the CHT, thus resembling the patient´s clinical profile and in contrast with what they observed in a cancer cell line with the same hormonal status. Consequently, the study highlighted the importance of using patient models in metastasis research [37]. In that same year, Lin et al. established a multiple myeloma zPDX through the injection of cells into the perivitelline space and observed a response-resistance correlation between patients and the zPDXs [146]. Similarly, Wu et al. detected angiogenesis, penetration of vasculature into the tumor and metastasis in the brain, trunk, and tail in a pancreatic zPDX model and observed a correlation between the model and the patients´ therapeutic response. In addition, they quantified human cell growth by cell dissociation and fluorescent counting, instead of imaging and fluorescent density measurement, which is the standard methodology to image tumor cell growth in embryos [144]. Along these lines, Al-Samadi et al. have proposed using quantitative PCR and droplet digital PCR, based on the human housekeeping gene GADPH instead of imaging to more precisely determine drug efficacy and dose-dependent responses [147].

Regarding the tumor microenvironment and zPDX, Wang et al. described an optimized and reliable pancreatic cancer zPDX with fibroblasts as a more robust in vivo model to assess the response of candidate drugs. First, cancer cells and fibroblasts were enriched from freshly-harvested or frozen pancreatic cancer tissue. Cells were then labeled with fluorescent reporter lentiviruses that also expressed the anti-apoptosis gene BCL2L1 in order to monitor the cell population in vivo and enhance cell viability, respectively. Mixed cancer cells and fibroblasts were injected into the yolk sac of 48 hpf embryos and treated with gemcitabine and/or navitoclax. Using their approach, the authors were successful in establishing zPDXs of pancreatic cancer that mimicked the tumor microenvironment and assessing drug response in both groups of cells. Furthermore, they proposed the additional co-injection of different human immune cells to achieve a more humanized microenvironment in future zPDX experiments [141]. In 2019, Sun et al. co-injected two prostate cancer cell lines with human-isolated CAFs into the yolk sac and demonstrated that fibroblasts promoted the proliferation and migration of prostate cancer cells through a TGF-β-mediated pathway paracrine effect. Moreover, when a TGF-β receptor inhibitor was added, cell proliferation and metastasis were significantly reduced, highlighting a new potential therapeutic target [148]. Similarly, Ren et al. showed that the fibrogenic activation of CAFs, through the TGF-β pathway, promoted breast cancer cell intravasation and extravasation when they co-injected both type of cells in the perivitelline space or the duct of Curvier [149]. Recently, Seoane et al. published a study unraveling how TAMs may affect breast cancer progression. Using in vitro assays and co-injection of breast cancer cells with macrophages into the yolk sac of 48 hpf zebrafish embryos, the authors were able to demonstrate that the overexpression of a transcription factor (POU1F1) modulates the tumor microenvironment and leads to the recruitment of macrophages and the differentiation of these cells into TAMs. In turn, the collaboration between cancer cells and TAMs further increases tumor proliferation [70].

The information derived from orthotopic and zPDX models and co-injection strategies highlights again the importance of the tumor microenvironment in cancer behavior, suggesting that a more extensive use of these approaches might lead to promising future developments in the individualized/personalized cancer therapy front. Nevertheless, cancer is, as stated before, a set of genetically diverse diseases, so genetic models in zebrafish ought to be considered in order to assess the implication of different genes in cancer phenotypes and their microenvironment. While this review does not focus on zebrafish genetic cancer models, we refer the reader to the following excellent reviews by Kirchberger et al., Astone et al., or Hason et al. [18,157,158].

## 4. The Future of the Zebrafish Xenografts Is Already Here

**Figure 2:**
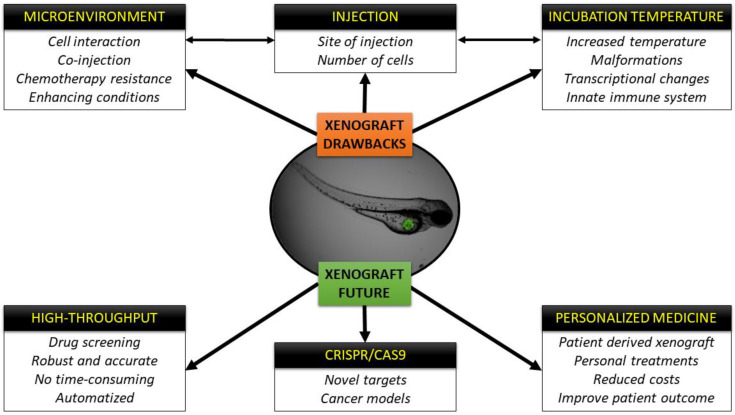
Schematic representation of the drawbacks of the xenograft technique and future perspectives and fields of application.

Xenograft assays in zebrafish embryos have been used in the cancer field for different purposes: from modeling cancer cells of diverse tumor entities, to assessing tumor cell proliferation [52,58], dissemination, or their invasive capacity [32]; to testing their angiogenic potential [29] or how new chemotherapeutic compounds influence their metabolism [33]. While xenografts have traditionally been performed in immunocompromised mouse models with tremendous success, small animal models are costly and studies with large numbers of mice to achieve statically significant differences go against the concept of the 3Rs: Replacement, Reduction and Refinement. The zebrafish xenograft model is an excellent alternative as discussed throughout this review, and while no model is perfect and each has its own inherent disadvantages, zebrafish have definitely made a splash in the cancer field due to the numerous benefits associated with this species (Figure 2). First of all, transparency allows the user to track cancer cells in real time, follow their proliferation depending on the site of injection [33,159], assesses processes like EMT or MET that takes place when the cells intravasate or extravasate the vasculature in order to establish a new metastatic niche, normally in the CHT of the embryos [160], or even co-inject macrophages or fibroblasts from the human microenvironment along with cancer cells to assay the interaction between them and their collaborative capacity to reform tumors in a new host [70]. In addition, the generation of transgenic lines with fluorescent vasculature in order to study tumor angiogenesis [39] or fluorescent macrophages or neutrophils to test the recruitment and reaction/interaction of the innate immune system with the injected human cells are powerful complementary tools that are available to researchers [84]. Likewise, the high number of off springs allows researchers to perform high-throughput screenings of different compounds [64,133], for example, clinically approved chemotherapies, combinations of treatments or novel compounds in a fast and low-cost way. Moreover, the automation of these screenings by means of automated injection, automated image acquisition; processing and comparison of images represent additional benefits that facilitate the work of researchers in this field [161]. Finally, the ease by which the completely sequenced zebrafish genome can be genetically manipulated [162] makes this model species a perfect candidate for Crispr/Cas9 editing [163,164]. The discovery and rapid implantation of genome modifications via Crispr/Cas9-based methodologies caught the attention of the zebrafish community, and led to the initiation of global efforts to generate new cancer zebrafish models (knock-down and knock-in) as a means of identifying new targets for drug development purposes [165].

The sum of these advantages and techniques (Figure 2) will surely have an impact on personalized medicine in the near future, where zebrafish avatar models could be incorporated into the clinical setting to facilitate personalized treatments for each patient with reduced costs and at speeds that rival other avatar models [13]. There is no doubt that the zebrafish xenograft model has its drawbacks and further improvements need to be made and are being explored, but we foresee in the not so far off future that zebrafish xenografts will likely play in important role in the how the future of cancer therapy evolves.

## Figures and Tables

**Table 1:** Summary of incubation temperatures in xenograft assays.

Temperature	Incubation Time	Injection Site	Time of Injection	Cell Line	Number of Cells Injected	Reference
28 °C	1–2 days	Perivitelline space	48 hpf	FGF2-T-MAE, Tet-FGF2, A2780, MDA-MB-435 and B16-BL16	4–10 nL	[29]
28 °C	4 days	Perivitelline space	48 hpf	BT549	n/a	[62]
28.5 °C	3 days	Yolk sac	48 hpf	ES2 and OV90	100–200	[63]
32 °C	3 days	Yolk sac	48 hpf	K562	100–200	[64]
32 °C	2 days	Perivitelline space	48 hpf	A549	100	[65]
33 °C	6 days	Blastodisc/Duct of Cuvier	Early blastula/48 hpf	hPSC-derived ECs	100/400	[66]
33 °C	3 days	Duct of Cuvier	48 hpf	HT29	200	[67]
34 °C	4 days	Duct of Cuvier	48 hpf	FGF-T-MAE, 4T1, MDA-MB-231, PC3, MAE and ZF4/PAC2	50–400	[39]
34 °C	7 days	Yolk sac	48 hpf	BT-474, MCF7 and MDA-MB-435	500	[52]
34 °C	6 days	Yolk sac	48 hpf	PC3, LNCAP, MCF7, BT474, A549, H460, H1299, HT29, SW620, MV3 and HT1080	100	[53]
34 °C	3 days	Yolk sac	48 hpf	TC252 and A673	500	[54]
34 °C	6 days	Yolk sac	48 hpf	Primary tumors (92.1 and Mel270) and UM metastases (OMM1, OMM2.3, OMM2.5).	400–500	[55]
34 °C	4 days	Duct of Cuvier	48 hpf	MDA-MB-231	300	[56]
35 °C	3 days	Yolk sac	48 hpf	MDA-MB-231	n/a	[59]
35 °C	1 day	Duct of Cuvier	48 hpf	Nalm-6 cells and CAR T cells	50–300	[60]
35 °C	3 days	Yolk sac	48 hpf	HCT 116, Mia Paca-2 and cancer tissue	n/a	[61]
35° C	3 days	Yolk sac	48 hpf	K562 and NB-4	25–50	[68]
35° C	2–4 days	Yolk sac	48 hpf	Jurkat, Karpas45, and TALL1 and patient samples	50–100/500	[69]
34 °C and 36 °C	3 days	Yolk sac	48 hpf	HCT116	100–200 or 400–500	[58]

**Table 2:** Notable zebrafish patient-derived tumor xenograft (zPDX) assays.

Tumor Type	Nº of Patients	Sample Collection	Zebrafish Line	Nº of Cells	Site of Injection	Stage	T	Remarkable Results	Reference
Colon, gastric, and pancreatic ductal adenocarcinoma	6 (2 of each)	Surgery	WT	Tissue	Yolk sac	Larvae- 48 hpf	35 °C	Cancer tissue survives and invade the yolk sac	[61]
Pancreatic, colon and gastric cancers	24 (12,8,4 respectively)	Surgery	WT	Tissue	Yolk sac	Larvae- 48 hpf	35 °C	Chemo sensitivity assays in agreement with clinical studies	[61]
Pancreatic cancer	3	Surgery	WT/Casper	50-80	Yolk sac	Larvae- 48 hpf	32 °C	Lentiviral use to prolong the observation window	[141]
Acute Leukemia	7	Bone marrow	WT	200–500	Pericardial space	Larvae- 48 hpf	32.5 °C	Correlation between differential cell dissemination and clinical outcome	[150]
Head and neck cancer	1	Surgery	WT	1000	Perivitelline space	Larvae- 48 hpf	34 °C	Tumor response evaluation through PCR	[147]
Hepatocellular carcinoma	13	Surgery	WT	200	Yolk sac	Larvae- 48 hpf	28–37 °C gradient	Tumor response to established drugs	[151]
Glioblastoma, Rhabdomyosarcoma, metastatic melanoma, breast cancer	6	Surgery/ Blood	Casper, *prkdc-/-,il2rga-/-*	5 × 10^5^	Intraperitoneal	Adult	37 °C	PDX engraftment/Zebrafish reared at 37º	[152]
Gastric cancer	14 (5 failed)	Surgery	*Tg(fli-1:EGFP)*	600–800	Yolk sac	Larvae- 48 hpf	32 °C	Angiogenesis, metastasis/Correlation between zPDX and clinic result	[144]
Adenoid cystic carcinoma of the salivary gland	2	Surgery	*Tg(kdrl:grcfp)*	100–200/ Tissue	Yolk sac/Precardiac sinus	Larvae- 48 hpf		CR PDX-cell cultures preserve tumor biology, metastatic behavior, and drug response	[153]
Colorectal cancer	10	Surgery	WT/*Tg(fli:EGFP)*	Cell suspension (5) Tissue (5)	Perivitelline space	Larvae- 48 hpf	34 °C	Proliferation, angiogenesis, and histological and treatment correlation with relapse and mutational status	[34]
Abdominal liposarcoma	1	Surgery	*Tg(kdrl:mCherry)*	50–400	Heart cavity	Larvae- 48 hpf	34 °C	Cell ability to survive and migrate	[154]
Pituitary tumor	2	Surgery	*Tg(fli1a:EGFP)*	100 (derived from spheres)	Sub-epidermal space (close to the SIV plexus)	Larvae- 48 hpf	32 °C	Isolation of progenitor/stem cells from patient-derived spheres/Invasive and angiogenic behavior	[155]
Multiple myeloma	6	Plasma	Casper	50–200	Perivitelline space	Larvae- 48 hpf		Cell growth/Response-Resistance correlation	[146]
Pituitary adenoma and NET	8 (2 failed)	Surgery	*Tg(fli1a:EGFP)*	100	Perivitelline space	Larvae- 48 hpf	32 °C	Angiogenesis, invasive behavior, and migration	[156]
Bone metastasis from breast cancer	1	Surgery	*Tg(fli1a:GFP)*	50–400	Duct of Cuvier	Larvae- 48 hpf	34 °C	Patient and primary cells behavioral correlation but not with a cell line	[37]
T-cell acute lymphoblastic leukemia	2	Bone marrow	Casper	500	Yolk sac	Larvae- 48 hpf	35 °C	Differential response to treatment in correlation to mutational status	[69]

## Abbreviations

CAFs	Cancer associated fibroblasts
CHT	Caudal hematopoietic tissue
CSCs	Cancer stem cells
CTCs	Circulating tumor cells
CR	Conditional reprogrammed
Crispr/Cas9	Clustered regularly interspaced short palindromic repeats/Cas9
CSF-1	Colony stimulating factor 1
CSF1R	Colony stimulating factor 1 receptor
dpi	Days post-fertilization
EGFR	Epidermal growth factor receptor
EGF	Epithelial growth factor
EMT	Epithelial-mesenchymal transition
ECM	Extracellular matrix
FAP	Fibroblast-activating protein
GPF	Green fluorescent protein
hpf	Hours post-fertilization
hpi	Hours post-injection
IGF-1	Insulin-like growth factor
IFN-γ	Interferon-γ
IL	Interleukin
M-CSF	Macrophage stimulation factor-1
MET	Mesenchymal-epithelial transition
NET	Neuroendocrine tumors
NO	Nitric oxide
PDX	Patient-derived xenograft
RFP	Red fluorescent protein
SIVs	Sub-intestinal vessels
TEMs	Tie2-expressing macrophages
TGF-β	Transforming growth factor- β
TAMs	Tumor associated macrophages
VEGF	Vascular endothelial growth factor
WT	Wild-type
zPDX	Zebrafish patient-derived xenograft
